# Combined analysis of expression data and transcription factor binding sites in the yeast genome

**DOI:** 10.1186/1471-2164-5-59

**Published:** 2004-08-26

**Authors:** Vijayalakshmi H Nagaraj, Ruadhan A O'Flanagan, Adrian R Bruning, Jonathan R Mathias, Andrew K Vershon, Anirvan M Sengupta

**Affiliations:** 1 BioMaPS Institute, Rutgers University, Piscataway, NJ 08854, USA; 2 Department of Physics and Astronomy, Rutgers University, Piscataway, NJ 08854, USA; 3 Waksman Institute and Department of Molecular Biology and Biochemistry, Rutgers University, Piscataway, NJ 08854, USA; 4 Medical Sciences Center, University of Wisconsin, Madison, WI 53706, USA

## Abstract

**Background:**

The analysis of gene expression using DNA microarrays provides genome wide profiles of the genes controlled by the presence or absence of a specific transcription factor. However, the question arises of whether a change in the level of transcription of a specific gene is caused by the transcription factor acting directly at the promoter of the gene or through regulation of other transcription factors working at the promoter.

**Results:**

To address this problem we have devised a computational method that combines microarray expression and site preference data. We have tested this approach by identifying functional targets of the **a**1-*α*2 complex, which represses haploid-specific genes in the yeast *Saccharomyces cerevisiae*. Our analysis identified many known or suspected haploid-specific genes that are direct targets of the **a**1-*α*2 complex, as well as a number of previously uncharacterized targets. We were also able to identify a number of haploid-specific genes which do not appear to be direct targets of the **a**1-*α*2 complex, as well as **a**1-*α*2 target sites that do not repress transcription of nearby genes. Our method has a much lower false positive rate when compared to some of the conventional bioinformatic approaches.

**Conclusions:**

These findings show advantages of combining these two forms of data to investigate the mechanism of co-regulation of specific sets of genes.

## Background

A bioinformatic approach to identifying cis-regulatory elements controlling transcription has become feasible with the availability of complete genome sequences and large scale expression data using high-throughput methods such as microarrays [1,2] and SAGE [3]. The expression data provides a list of genes whose expression is significantly modified under a particular condition. However, this data does not indicate whether these genes are direct targets of a particular transcription factor or if the changes in expression are the result of an indirect effect caused by altering the expression of other transcription factors that work directly at the promoter. Using information about sequence preference for binding of particular transcription factors, one can identify possible regulatory binding sites within a sequenced genome. However, this approach does not indicate if the sites are functional. We have therefore developed an algorithm that combines both of these approaches to distinguish between the direct and indirect targets that are regulated by a particular transcription factor.

We have applied this methodology to study the transcriptional regulatory system that specifies cell mating-type in the yeast *Saccharomyces cerevisiae *[4]. Yeast have three cell types, haploid **a **and *α *cells, and the **a**/*α *diploid, that differ in their ability to mate and in the proteins they express. Cell mating-type is determined in part by *α*2 and **a**1, which are cell-type-specific proteins that are members of the homeodomain (HD) DNA-binding family. In an **a**/*α *diploid cell, *α*2 binds with **a**1 to form a heterodimer complex that represses transcription of haploid-specific genes [5]. The crystal structures of the *α*2 HD binding DNA alone and in complex with **a**1 have been solved, providing models for how these complexes bind DNA [6,7]. Biochemical and mutational analysis of each protein and their DNA-binding sites have defined the requirements for DNA recognition by this complex [8-10]. Genome-wide expression analysis has also been performed on each of the different cell types [11]. The combination of these resources has allowed us to develop and test algorithms to identify target sites for the **a**1-*α*2 complex. Previous work, using a relatively simple binding site search program identified targets for the *α*2-Mcm1 complex, which represses **a**-cell-type specific genes in *α *and **a**/*α *cells [12]. The more advanced methods described in this paper have helped identify several novel targets of the **a**1-*α*2 complex that may be involved in cell-type specific processes. Interestingly, we identified several genes that are repressed in diploid cells but do not appear to be direct targets of the **a**1-*α*2 complex, suggesting that these genes are controlled by another transcriptional regulatory factor that is directly or indirectly regulated by the **a**1-*α*2 complex. We have also identified a number of **a**1-*α*2 target sites that do not repress adjacent genes. The combination of site preference and expression data is therefore a valuable tool to identify direct functional targets of a transcription factor or complex.

## Results

### Development of a search algorithm for targets of the a1-*α*2 complex

To generate an algorithm that combines microarray expression data and mutational analysis of binding sites, we first defined a scoring method that ranks gene expression data. We utilized the microarray expression data from Galitski and coworkers for gene expression in the **a **and *α *haploid and **a/***α *diploid cells, as well as various polyploids [11]. Since the **a**1-*α*2 complex should be absent in any of the homozygous **a **or *α *type polyploids (**a, aa**, ..., *α,αα*,..., etc.) we expect the expression of haploid-specific genes in these cells to be much higher than in cells that are heterozygous for the *MAT *locus (**a***α*, **aa***α*, **a***αα*, **aa***αα*, etc.). Thus, one term in the scoring function rewards lower expression in heterozygous cell types compared to the homozygous cell types (see the Methods section for details). We expect that most of the haploid-specific genes will be expressed equally in both of **a **and *α *cell types. Consequently, we have introduced a second term in the scoring function that penalizes such differences in expression in the two haploid cell types. This scoring function would identify haploid-specific genes that are repressed in diploid cells, but would not indicate if these genes are direct targets of the **a**1-*α*2 repressor complex.

To identify genes from this ranking that are directly repressed by the **a**1-*α*2 complex we used the available mutational data on the **a**1-*α*2-binding site [9]. In these experiments, the effects of single base pair mutations of the **a**1-*α*2 consensus binding site were measured by assaying their ability to repress transcription of a heterologous promoter and by electrophoretic mobility shift DNA-binding assays (EMSA). Under the assumption that the level of expression is proportional to how often that site is unoccupied, we used the effects of the single base mutations to estimate the parameters for the binding energy of sites with different bases at each position. We then used this information to search for potentially strong binding sites in the promoter regions (in practice, 800 bp upstream of the translation start site) of every gene in the genome. This search provides us with a list of genes with putative **a**1-*α*2 binding sites in the promoter, irrespective of functionality.

Even under ideal conditions, either of the above lists of genes would not directly indicate a haploid-specific gene directly repressed by the **a**1-*α*2 complex. In addition, our accuracy is limited by the noise in the microarray expression data, as well as by simplifying assumptions made to utilize single-base mutational data to score arbitrary sequences. We therefore decided to rank the genes using a scoring system that takes into account expression patterns across different mating types, as well as the likelihood of finding a good **a**1-*α*2-binding site in the promoter region of the gene. To test the significance of these composite scores, we generated permuted data, which combined random promoters with the expression data. This analysis suggested that the top 10–15 predictions were significant. The results of experiments on the 24 genes with the highest combined scores from our analysis are shown in Table 1.

**Table 1:** Potential **a**1-*α*2 Binding Sites in Haploid-specific Genes

ORF	Gene	Expression p-val^a^	Binding p-val^b^	Combined p-val	**a**1-*α*2 ChIP^c^
*YDL227C*	*HO*	0.0006	0.0017	1.1e-6	+^f^
*YLR265C*	*NEJ1*	0.0003	0.0053	1.7e-6	+
*YBL016W*	*FUS3*	0.0001	0.0991	1.6e-5+	
*YOR212W*	*STE4*	0.0020	0.0082	1.7e-5	+
*YJR086W*	*STE18*	0.0008	0.0218	1.7e-5	+
*YHR005C*	*GPA1*	0.0005	0.0437	2.1e-5	+
*YDR103W*	*STE5*	0.0017	0.0298	5.2e-5	+
*YBR073W*	*RDH54*	0.0053	0.0116	6.2e-5	+
*YGR044C*	*RME1*	0.0009	0.0720	6.8e-5	+^f^
*YGL248W*	*PDE1*	0.0182	0.0040	7.3e-5	+
*YPL038W*	*MET31*	0.0292	0.0027	8.0e-5	+
*YDR088C*	*SLU7*	0.0303	0.0038	1.2e-4	-
*YGL052W*		0.0117	0.0109	1.3e-4	-
*YJL157C*	*FAR1*	0.0013	0.1141	1.4e-4	+
*YPR122W*	*AXL1*	0.0091	0.0163	1.5e-4	+^f^
*YIL099W*	*SGA1*	0.0063	0.0267	1.7e-4	-
*YLR233C*	*EST1*	0.0226	0.0090	2.0e-4	-
*YKL182W*	*FAS1*	0.0578	0.0035	2.1e-4	-
*YMR053C*	*STB2*	0.0028	0.0884	2.5e-4	-
*YNL319W*		0.0123	0.0222	2.7e-4	-
*YFR012W*		0.0088	0.0125	2.8e-4	-
*YNL188W*	*KAR1*	0.0019	0.1890	3.6e-4	-
*YGL193C*		0.0014	0.2654	3.8e-4	-
*YMR157C*	*FMP39*	0.1557	0.0026	4.0e-4	-
*YBR158W*^d^	*AMN1*	0.0037	0.0221	8.7e-5	+
*YCL066W*^e^	*MATα1*	0.1630	0.9510	1.5e-1	+^f^

^a ^The ranking of haploid-specific gene expression determined by analysis of microarray data [11].

^b ^The ranking of potential **a**1-*α*2 target of haploid-specific genes.

^c ^A + indicates that the **a**1-*α*2 binds to the promoter by ChIP assay.

^d ^Identified in a search for sites with 1500 bp from the start of the ORF

^e ^Identified if remove the penalty for expression in one haploid cell type but not the other.

^f ^Identified as direct targets of the **a**1-*α*2 repressor complex in previous studies.

### **a**1-*α*2 binding to the promoter regions of genes identified in the search

**Figure 1:**
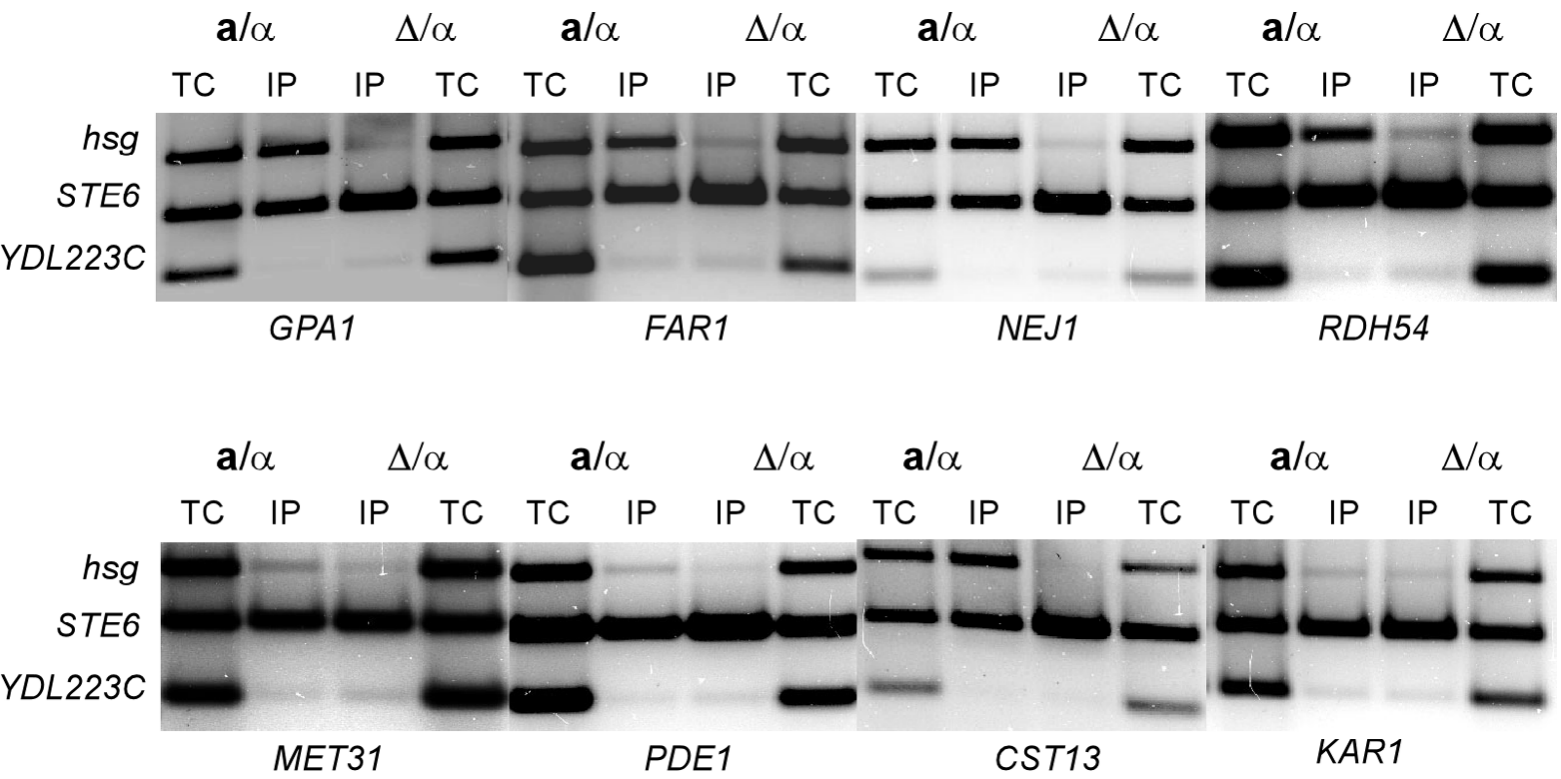
ChIP assays of promoter fragments that are predicted to be targets of the **a**1-*α*2 complex. ChIP assays with antibody to the *α*2 protein were performed on lysates from *MAT**a***/*MATα *(**a**/*α*) and *mat**a***Δ/*MATα*(Δ/*α*) cells. Total chromatin (TC) and immunoprecipitated (IP) samples were subjected to multiplex PCR with primers flanking potential **a**1-*α*2 sites (labeled *hsg*) in the indicated promoters. Primers for the promoter region of *STE6*, an **a**-specific gene that is repressed by the *α*2-Mcm1 complex in both cell types were used as a positive control for the ChIPs. Primers that hybridize in *YDL223C*, a gene that is not regulated by *α*2, was used as the negative control. The presence of a band over background levels from the test promoter from **a**/*α *lysates but not *mat*Δ/*α *indicates that the **a**1-*α*2 complex is specifically binding to the promoter. Assays for the *GPA1, FAR1, NEJ1, RDH54, MET31, PDE1, AMN1/CST13 *and *KAR1 *are shown. A summary of the all of the ChIPs performed is in Table 1.

To evaluate the success of our computational algorithm for identifying direct targets of the **a**1-*α*2 repressor complex, we assayed binding by the complex to the identified promoters using chromatin-immunoprecipitation (ChIP) assays with polyclonal antibody directed against the *α*2 protein. In *α *haploid and **a/***α*-diploid cells the *α*2 protein combines with the MADS-box transcription factor Mcm1 to bind to elements in the promoters of **a**-specific genes to repress their transcription [13,14]. We therefore included in each PCR a primer set for the promoter region of the **a**-specific gene *STE6 *to serve as a positive control for the ability to ChIP *α*2 in both haploid *α *and diploid **a/***α *cells. This primer set also allowed us to rule out the possibility that the predicted **a**1-*α*2 target genes were immunoprecipitating because of binding by the *α*2-Mcm1 complex. The primer set for the *YDL223C *promoter, a gene not bound or repressed by the **a**1-*α*2 or *α*2-Mcm1 complexes, was included in the reaction as a negative control for non-specific immunoprecipitation of the DNA. A gel displaying the results for a few of the promoters that were assayed is shown in Figure 1 and the data summarized for all the promoters that were predicted to be directly repressed by the **a**1-*α*2 complex is listed in Table 1. In general, there is a very good correlation between the experimental data with the predictions based on our computational algorithm. Almost all of the high scoring genes were bound by the **a**1-*α*2 complex in vivo. Genes that had a combined p-value higher than the threshold of 1.5 × 10^-4 ^(which corresponds to choosing the top 15 of the list in Table 1) do not appear to be strongly bound by the complex. This p-value corresponds to roughly a probability of one in six thousand, indicating it is possible to get such combination by chance.

In comparison to higher eukaryotes, most yeast promoters are relatively small and contain activator or repressor binding sites within several hundred base pairs of the start site of the open reading frame (ORF) of the gene. However, there are a few genes, like *HO*, whose regulation is controlled by a region several Kb long. Consequently, we did a separate search looking for additional **a**1-*α*2 binding sites that are within a region 1.5 Kb upstream of the ORF. Most of the sites identified in this search were well above the threshold value and were not bound by the **a**1-*α*2 complex in ChIP assays (data not shown). However, the search identified one site upstream of the *AMN1/CST13 *gene that was a potential target site. The ChIP analysis verified this as a functional target site for the **a**1-*α*2 complex in vivo (Figure 1, Table 1).

### Analysis of haploid-specific genes predicted not to be bound by **a**1-*α*2

**Figure 2:**
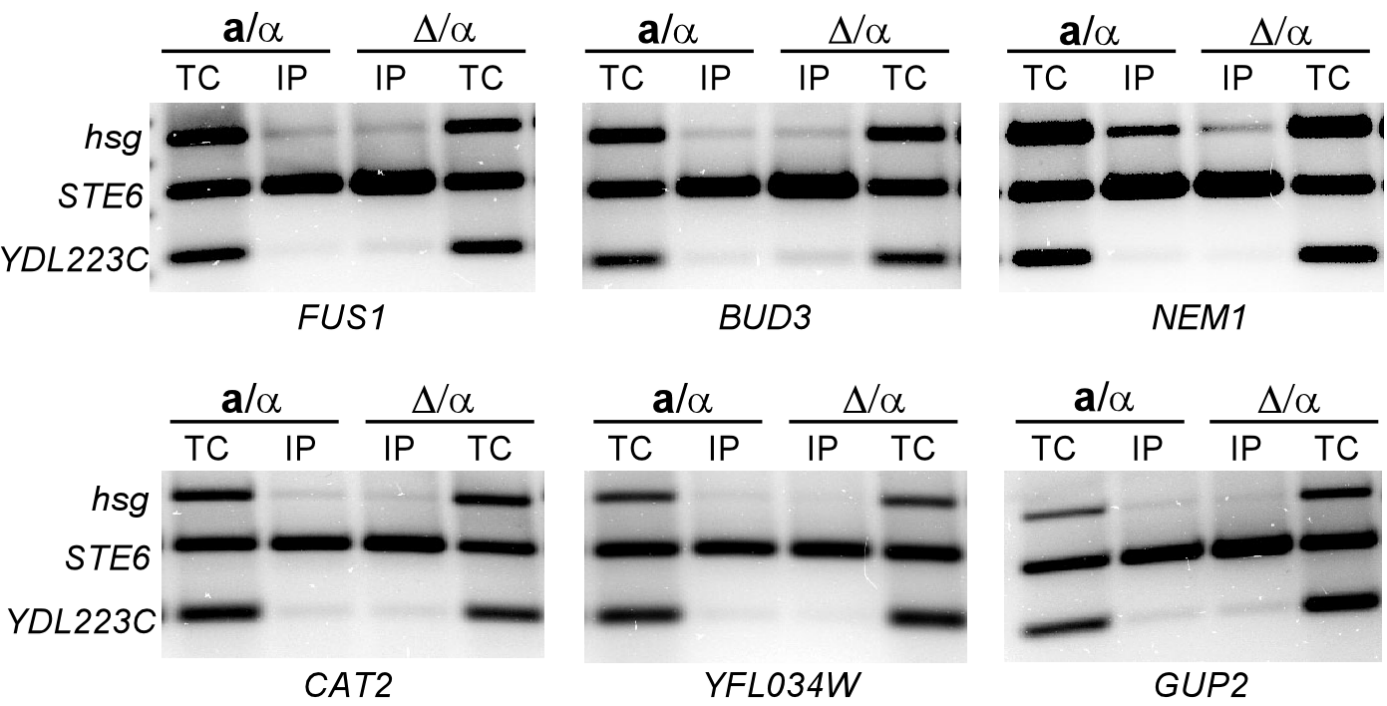
ChIP assays of **a**1-*α*2 binding to promoter fragments that are not predicted to be targets of the complex. ChIP assays were performed as described in Figure 1. ChIP assays for **a**1-*α*2 binding to the *FUS1*, *BUD3*, *NEM1*, *CAT2*, *YFL034W *and *GUP2 *promoters are shown.

Among the top 35 genes in the list ranked by haploid-specific expression score, more than half do not appear to contain an identifiable **a**1-*α*2 site by our analysis (lack of a significant binding site being defined as binding p-value greater than 2 × 10^-4^) (Table 2). Although there were no apparent strong affinity **a**1-*α*2-binding sites in the promoters of these genes, it is possible that there are several weak affinity sites in the promoter that were not identified in the search. If present, these sites may work cooperatively to increase binding by the **a**1-*α*2 complex to the promoter and therefore repress transcription. In support of this model we have found that under some conditions weak affinity **a**1-*α*2 sites in the *HO *promoter have a role in repression of the promoter (Mathias and Vershon, unpublished). Promoters with a number of weak affinity sites may therefore be directly regulated by the **a**1-*α*2 complex. However, it is also possible that these genes are indirectly repressed by **a**1-*α*2, through its ability to repress expression of another transcription factor that is required for expression of the genes identified in the microarray. To distinguish between these possibilities we assayed for **a**1-*α*2 binding to these promoters by ChIP (Figure 2 and Table 2). As predicted from the site identification analysis, the **a**1-*α*2 complex did not appear to bind to most of these promoters. This result suggests that these genes are not directly repressed by the complex. The one exception to our predictions was that the **a**1-*α*2 complex appeared to weakly bind to the *NEM1 *promoter in the ChIP assays (Figure 2). Interestingly, *NEM1 *is downstream of *GPA1*, a gene that is strongly repressed by the **a**1-*α*2 complex (Fig 1). Binding and repression by **a**1-*α*2 at *GPA1 *may help binding to weak sites in the *NEM1 *promoter. Alternatively, although we sheared the DNA used in the ChIP to an average of less than 500 bp, it is possible that there may have been some fragments that spanned the ~2 kb between the genes, thereby giving a positive result in the ChIP assay.

**Table 2:** Haploid-specific Genes that Do Not Contain **a**1-*α*2 Target Sites

ORF	Gene	Expression p-val^a^	Binding p-val^b^	Combined p-val	**a**1-*α*2 ChIP^c^
*YIL117C*	*PRM5*	0.0011	0.3690	0.0004	-
*YLR159W*		0.0015	0.4050	0.0006	-
*YBR051W*		0.0022	0.4390	0.0010	-
*YLR080W*	*EMP46*	0.0024	0.6757	0.0016	-
*YIR039C*	*YPS6*	0.0025	0.7843	0.0020	-
*YCL014W*	*BUD3*	0.0027	0.3475	0.0009	-
*YPL189W*	*GUP2*	0.0030	0.3708	0.0011	-
*YJL077C*	*ICS3*	0.0032	0.9540	0.0030	-
*YML042W*	*CAT2*	0.0033	0.7564	0.0025	-
*YHR004C*	*NEM1*	0.0035	0.6396	0.0022	+
*YFR046C*	*CNN1*	0.0036	0.5712	0.0020	-
*YDR220C*		0.0038	0.5919	0.0022	-
*YPL025C*		0.0041	0.6844	0.0028	-
*YBR006W*	*UGA2*	0.0043	0.7684	0.0033	-
*YBR108W*		0.0044	0.4390	0.0019	-
*YFL034W*		0.0046	0.3287	0.0015	-
*YCL027W*	*FUS1*	0.0047	0.7170	0.0034	-
*YLR308W*	*CDA2*	0.0049	0.3424	0.0017	
*YGR014W*	*MSB2*	0.0050	0.6554	0.0033	
*YJL202C*		0.0052	0.6416	0.0033	

^a ^The ranking of haploid-specific gene expression determined by analysis of microarray data [11].

^b ^The ranking of potential **a**1-*α*2 target of haploid-specific genes.

^c ^A + indicates that the **a**1-*α*2 binds to the promoter by ChIP assay.

### Comparison with binding site identification by the weight matrix method

We compare the performance of our algorithm with that of the weight matrix method [15-18]. In our study, we derived our parameters from a set of artificial sequences. Usually, the weight matrix has to be constructed from a set of known sites. We calculate the weight matrix for **a**1-*α*2 from regulatory elements upstream several known target genes: *HO, GPA1, FUS3, AXL1, STE5, RME1 *and *MATα1*. As usual, one is faced with a choice of threshold weight matrix score for selecting putative sites in the yeast genome. For a stringent threshold that corresponds to the top 16 targets, we recovered all the genes, other than *RME1*, used in construction of the weight matrix. However, we did not recover most of the other genuine targets identified, and verified, in this study. If we set the threshold to be lax enough to include *RME1*, we obtained 55 candidate genes, including *STE18 *and *RDH54, *but still miss targets like *STE4*. It is likely that most of the 55 putative targets are false positives, as evidenced by lack of haploid-specific regulation in the corresponding gene expression data.

Overall, we find our method to be more successful than the weight matrix method. The use of mutational data as opposed to literature based data for sequence preference possibly accounts for part of the success (an advantage we may not have for some other transcription factors). However, much of our success has to do with cutting down of false positive rates by using microarray data judiciously.

### Analysis of all potential **a**1-*α*2 target sites in the genome

**Figure 3:**
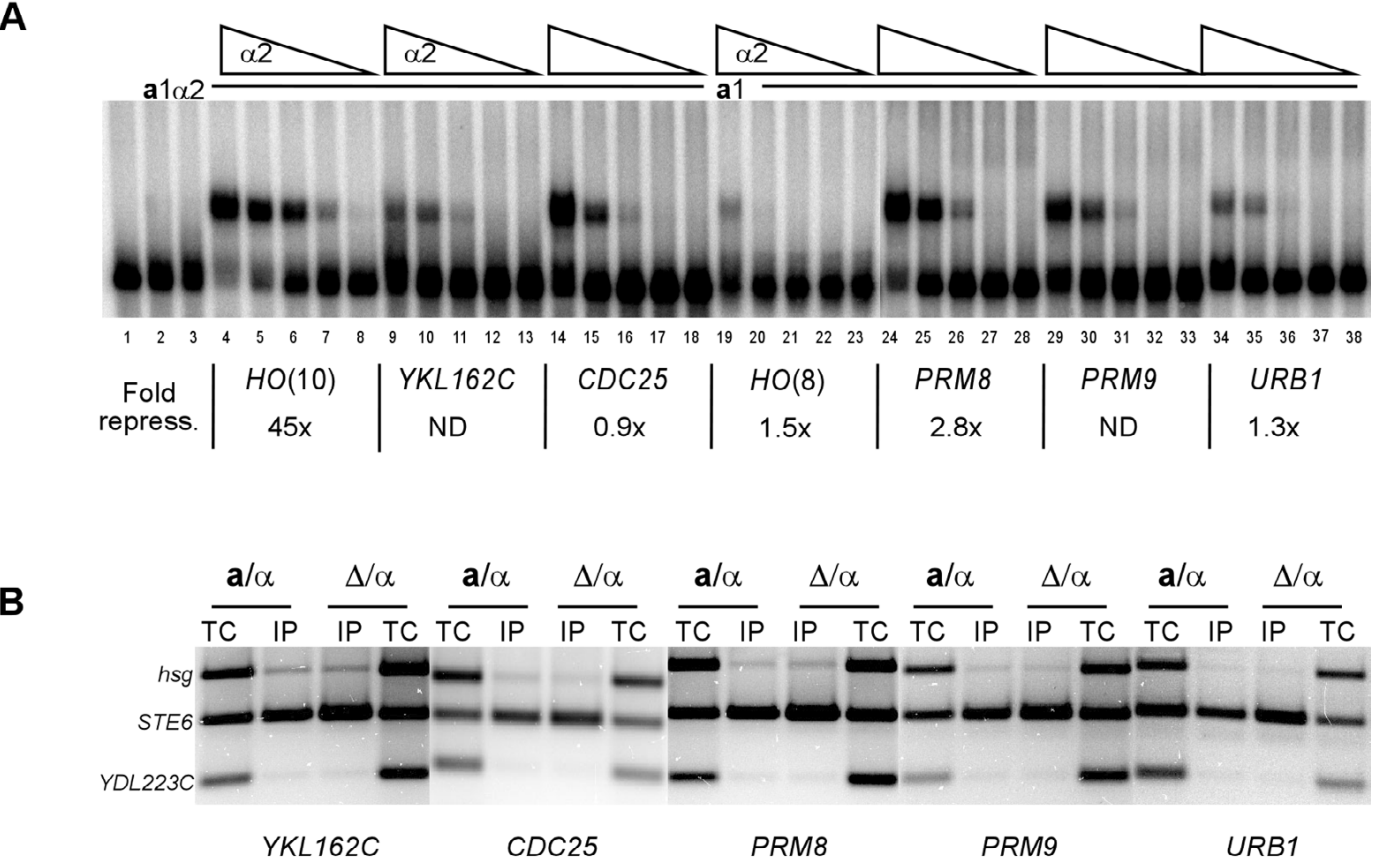
**a**1-*α*2 binding in vitro and in vivo to sites in the ORF regions of the genome. (A) An EMSA of purified **a**1 and *α*2 proteins binding to a strong **a**1-*α*2 binding site, *HO(10) *(lanes 1–8) and sites from the *YKL162C *(lanes 9–13), *CDC25 *(lanes 14–18), a weak **a**1-*α*2 binding site from the *HO *promoter, *HO(8) *(lanes 19–23), *PRM8 *(lanes 24–28), *PRM9 *(lanes 29–33) and *URB1 *(lanes 34–38). The concentration of the **a**1 protein was held constant at 1.4 × 10^-6 ^M (lanes 2 and 4–38) and mixed with 5-fold dilutions of the *α*2 protein starting at 8.2 × 10^-8 ^M (lanes 3, 4, 9, 14, 19, 24, 29 and 34)). The EMSAs shown are phosphorimages of the gels. Lane 1 contains *HO(10) *probe alone, and lanes 2 and 3 contain 1.4 × 10^-6 ^M **a**1 and 8.2 × 10^-8 ^M *α*2 respectively. The fold repression by each site in the context of a heterologous promoter is shown below each gene/promoter. (B) ChIP assays for the genes *YKL162C*, *CDC25*, *PRM8*, *PRM9 *and *URB1 *are shown. ChIP assays were performed as described in Figure 1

Among the genes identified in the computational analysis, there is a good correlation between the presence of strong **a**1-*α*2-binding sites in their promoter region and repression in diploid cells. This raises the question of whether all **a**1-*α*2-binding sites function as repressor sites. To address this question we searched for all potential binding sites in the yeast genome. As expected, many of the best sites are in the promoters of known or previously identified haploid-specific genes (Table 1). However, we also identified a number of putative **a**1-*α*2-binding sites within ORFs (Table 3). To test if the **a**1-*α*2 complex is able to bind to these sites we performed electrophoretic mobility shift assays (EMSAs) with purified *α*2 and **a**1 proteins and radiolabeled oligonucleotides containing these sites (Fig 3A). The **a**1-*α*2 complex bound to sites from the *YKL162C*, *CDC25*, *PRM8*, *PRM9*, and *URB1 *ORFs with weaker affinity than to a strong binding site from the *HO *promoter, *HO(10)*. However, these sites did have slightly better binding affinity than to the *HO(8) *site, which we have shown is unable to repress transcription on its own (Mathias and Vershon, unpublished).

**Table 3:** Potential **a**1-*α*2 target sites in ORFs

ORF	Gene	Expression p-val^a^	Binding p-val^b^	Combined p-val	**a**1-*α*2 ChIP^c^	EMSA^d^
*YKL014C*	*URB1*	0.621	0.042	0.025	-	25×
*YGL053W*	*PRM8*	0.291	0.011	0.002	-	5×
*YAR031W*	*PRM9*	0.891	0.013	0.012	-	10×
*YKL162C*		0.782	0.017	0.014	-	10×
*YLR310C*	*CDC25*	0.102	0.018	0.002	-	5×
*YPL061W*	*ALD6*	0.597	0.018	0.009	-	
*YBR028C*		0.793	0.046	0.036	-	
*YOL022C*		0.563	0.023	0.013	-	
*YJR016C*	*ILV3*	0.248	0.025	0.006	-	
*YBR218C*	*PYC2*	0.324	0.027	0.008	-	
*YFR040W*	*SAP155*	0.234	0.165	0.038	-	
*YMR269W*		0.332	0.049	0.016	-	
*YJL129C*	*TRK1*	0.297	0.083	0.024	-	
*YCR053W*	*THR4*	0.607	0.073	0.044	-	

^a ^The ranking of haploid-specific gene expression determined by analysis of microarray data [11].

^b ^The ranking of potential **a**1-*α*2 target of haploid-specific genes.

^c ^A + indicates that the **a**1-*α*2 binds to the promoter by ChIP assay.

^d ^Relative binding affinity in fold decrease in affinity compared to the *HO(10) *binding site.

Since the **a**1-*α*2 complex was able to bind to these sites with weak to moderate affinity in vitro, it is possible these sites may partially repress transcription on their own. To test this model, we cloned these sites into the context of the *CYC1 *promoter driving expression of a *lacZ *gene and measured the ability of the sites to repress transcription of the reporter in diploid cells [9]. The sites from the *CDC25 *and *URB1 *ORFs did not repress transcription of the reporter promoter in diploid cells (Fig 3A). However, the site from *PRM8 *ORF, which showed the highest binding affinity among the sites found in ORF regions, weakly (2.8-fold) repressed the reporter promoter. This result indicates that this site can function as a repressor site in vivo if placed in the proper context. We next tested whether **a**1-*α*2 bound to these sites in the normal genomic context in vivo by ChIP assays. None of the sites in the ORF regions were bound by the **a**1-*α*2 complex (Fig 3B and Table 3). This result indicates that while they are competent for weak binding and repression in a heterologous promoter, they are unable to repress transcription in their normal genomic context.

**Figure 4:**
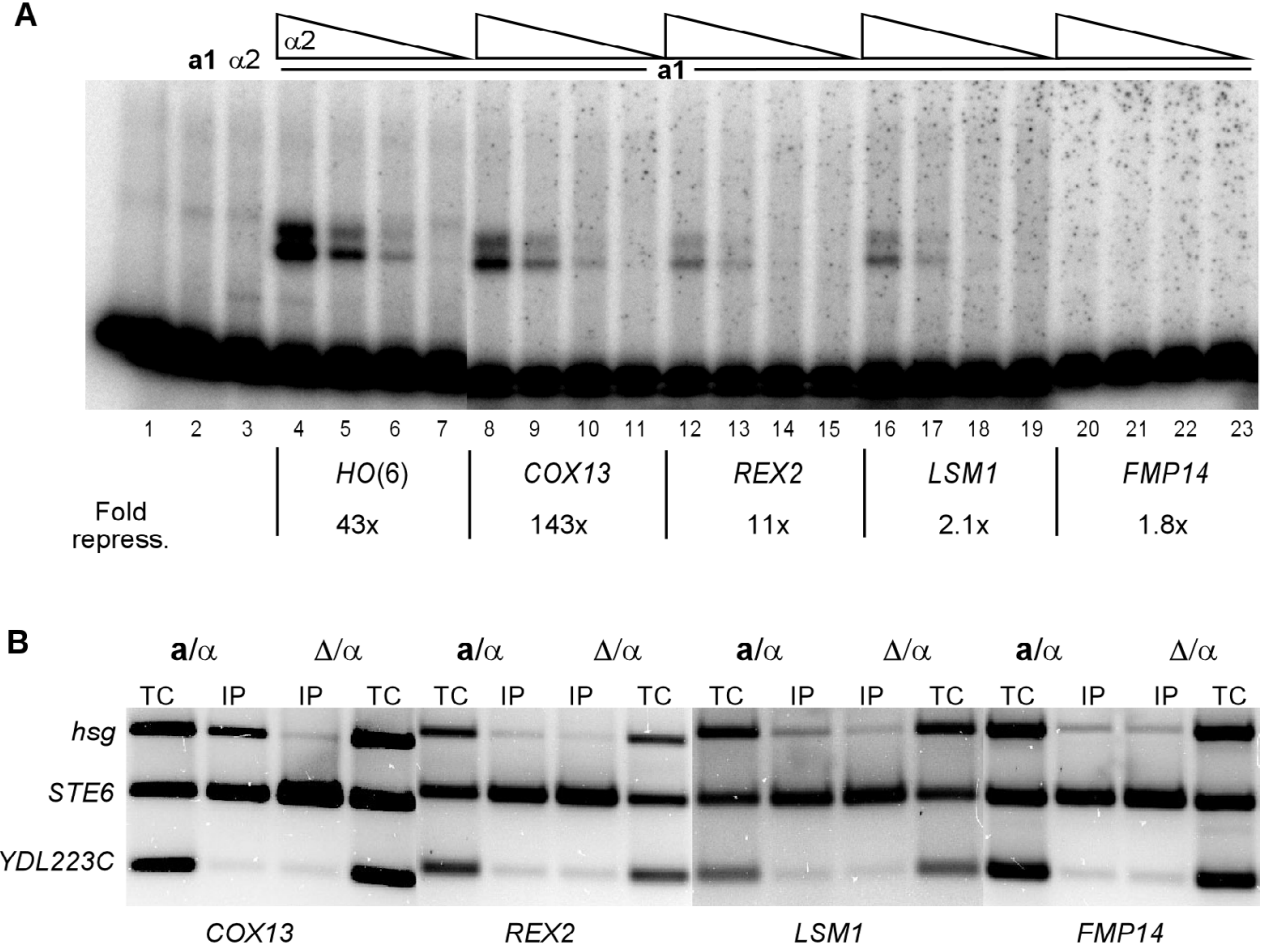
**a**1-*α*2 binding in vitro and in vivo to putative binding sites in promoters associated with genes which are not expressed in a haploid-specific manner. (A) An EMSA of purified **a**1 and *α*2 proteins binding to a strong **a**1-*α*2 binding site, *HO(6) *(lanes 1–7), *COX13 *(lanes 8–11), *REX2 *(lanes 12–15), *LSM1 *(lanes 16–19) and *FMP14 *(lanes 20–23). The concentration of the **a**1 protein was held constant at 1.4 × 10^-6 ^M (lanes 2 and 4–23) and mixed with 5-fold dilutions of the *α*2 protein starting at 8.2 × 10^-8 ^M (lanes 3, 4, 8, 12, 16 and 20). The EMSAs shown are phosphorimages of the gels. Lane 1 contains *HO(6) *probe alone, and lanes 2 and 3 contain 1.4 × 10^-6 ^M **a**1 and 8.2 × 10^-8 ^M *α*2 respectively. The fold repression by each site in the context of a heterologous promoter is shown. (B) ChIP assays for *COX13*, *REX2*, *LSM1 *and *FMP14 *were performed as described in Figure 1.

Our search also identified several potential **a**1-*α*2 binding sites in the promoter regions of genes that do not appear to be repressed in diploid cells (Table 4). Only the *COX13 *site had moderate binding affinity for the **a**1-*α*2 complex in the EMSAs (Fig 4A). However, despite the relatively weak binding affinity of these sites, they were able to partially repress transcription of the reporter in diploid cells (Fig 4A). In particular, the sites from the *COX13 *and *REX2 *promoters showed significant levels of repression. Interestingly, although these sites functioned as repressor sites in the context of the heterologous reporter, except for the *COX13 *promoter, most of these sites were not bound by the **a**1-*α*2 complex at their genomic locations by ChIP assays (Fig 4B). These results suggest that the genomic context of most of these **a**1-*α*2 sites prevents binding by the complex.

**Table 4:** Potential **a**1-*α*2 Target Sites in the Promoters of Non-Haploid-specific Genes

ORF	Gene	Expression^a ^p-val	Binding p-val	Combined p-val	**a**1-*α*2 ChIP^b^
*YGL191W*	*COX13*	0.4111	0.0053	0.0021	+
*YPL099C*	*FMP14*	0.1622	0.0075	0.0012	
*YLR059C*	*REX2*	0.1532	0.0098	0.0015	+/-
*YDR212W*	*TCP1*	0.5694	0.0017	0.0010	
*YJL124C*	*LSM1*	0.0830	0.0120	0.0010	+/-
*YAR033W*	*MST28*	0.7419	0.0133	0.0099	-
*YMR015C*	*ERG5*	0.6021	0.0138	0.0083	-
*YMR291W*		0.2659	0.0218	0.0058	-
*YPL188W*	*POS5*	0.2648	0.0230	0.0061	+/-
*YDR101C*	*ARX1*	0.3694	0.0298	0.0110	+
*YHR058C*	*MED6*	0.4507	0.0350	0.0157	
*YGL117W*		0.5577	0.0370	0.0206	
*YIL027C*	*KRE27*	0.5762	0.0381	0.0220	

^a ^The ranking of haploid-specific gene expression determined by analysis of microarray data [11].

^b ^A + indicates that the **a**1-*α*2 binds to the promoter by ChIP assay. A +/- indicates weak (2-fold) enhancement of the band in **a**/*α *cells by ChIP assay.

## Discussion

Genome-wide gene expression data using SAGE or DNA microarrays has provided a wealth of information on the regulation of genes under certain conditions or by specific transcription factors. The combination of this information with sequence analysis programs has enabled researchers to identify potential regulatory sites. For example, in a pioneering paper, Tavazoie *et al. *clustered expression data and used multiple local sequence alignment algorithms on the promoter regions of the co-clustered genes to discover regulatory motifs [19]. This approach has been further refined by using Bayesian networks to incorporate additional constraints regarding relative positions and the orientations of the motifs [20]. Another approach has been to break the genes into modules and perform module assignments and motif searches at the same time via an expectation maximization algorithm (as opposed to clustering first and finding motifs later) [21,22]. Although these approaches have worked well at identifying potential targets sites one drawback is that the expression patterns have to cluster well for these methods to work. For a small number of microarray experiments, this may always not be the case. A method that does not utilize clustering is a regression model based analysis to locate "words" in the promoter that correlates with modulation of expression [23]. However, this approach is restricted to retrieving functional consensus binding sites in the promoter regions and for transcription factors with low sequence specificity, this approach needs to be modified. Most of these approaches attack the difficult problem of what to do when relatively little is known about the regulatory system and sequence recognition by the protein. Consequently they develop pattern recognition algorithms that are essentially unsupervised. Our focus has been to take advantage, as much as possible, of knowledge about the biological system and use that information combined with expression analysis to identify potential target sites. The minor loss of generality of the tools resulting from such an approach is more than offset by its predictive power.

To determine if the changes in expression of a specific gene are the result of a transcription factor working at the promoter we developed an algorithm that combines expression data with information on the binding site preference for a transcription factor. As a test for this algorithm we identified genes in yeast that are direct targets for regulation by the **a**1-*α*2 repressor complex. We also used this method to identify genes that are repressed in diploid cells but that are not direct targets of the complex, as well as functional **a**1-*α*2 binding sites that do not appear to repress transcription in their genomic context. The combination of these sets of findings has provided insight into the regulatory network and mechanism of repression by the **a**1-*α*2 complex.

The primary goal of this study was to identify genes that are direct targets for repression by the **a**1-*α*2 complex. There are two major functional subsets among the **a**1-*α*2 target genes identified in this analysis (Table 1). One, not surprisingly, involves genes that are required for various processes in mating of the two haploid cell-types. These include components of the mating pheromone signal transduction pathway, such as *GPA1*, *STE18*, *STE4*, and *STE5, *which are activated in response to the binding of pheromone from the other cell type [24]. This group also includes genes further down that pathway, such as *FAR1 *and *FUS3*, which are required for cell-cycle arrest before mating. A number of these genes have previously been shown or suspected to be under the control of **a**1-*α*2 repressor complex [25,26]. Repression of these genes in diploid cells is biologically important because it prevents further mating by diploid cells. If diploid cells mate they would form triploids or higher ordered genomic polyploids, which are genetically unstable during meiosis and therefore detrimental to cell survival.

The second subset of genes identified in the analysis is associated with mating type switching and recombination. The *HO *gene is a known target of the **a**1-*α*2 complex and its promoter contains 10 binding sites of varying affinity [25]. Repression of *HO *is essential in diploid cells because it prevents switching of one of the *MAT *loci to form homozygous **a**/**a **or *α*/*α *diploid cells. Although diploid in genomic content, cells homozygous for the *MAT *loci are competent to mate and therefore would form higher order genomic polyploids that are genetically unstable. We have also shown that *NEJ1*, which is involved in non-homologous end-joining (NHEJ), is a direct target for the **a**1-*α*2 complex [27,28]. It has been proposed that that repression of the NHEJ pathway may promote homologous recombination and crossing over in diploid cells. In addition, we found that *RDH54*, a gene involved in double-stranded DNA break repair, is a direct target for the **a**1-*α*2 complex [29]. This result is somewhat unexpected because *RDH54 *is required for meiosis and null mutants show significantly reduced spore viability. It is likely that the **a**1-*α*2 complex only partially reduces the level of expression of the gene and that diploid cells require a lower level of activity of the protein.

We also identified several genes that fell outside of these two subsets. One is *RME1*, which encodes a transcriptional repressor of *IME1*, the master regulator of meiosis [30-32]. **a**1-*α*2-mediated repression of *RME1 *is required to allow cells to enter the meiotic pathway in diploid cells. Interestingly, we also found that *PDE1 *and *MET31 *are weakly, but reproducibly, direct targets for repression by the **a**1-*α*2 complex. The Pde1 protein is a low affinity cAMP phosphodiesterase that appears to have a role in response to stress and cell aging [33]. Repression of *PDE1 *in diploids may partially account for the difference of starvation response between haploids and diploids. Met31 is a zinc finger DNA-binding protein that activates genes involved in sulfur metabolism [34]. It is unclear why this gene would be a target for the **a**1-*α*2 complex.

It is possible that the presence of an **a**1-*α*2 target site upstream of a gene that has lower expression in diploid cells was fortuitous and that these sites were not functional targets. However, if this was the case then there would be little pressure to conserve these binding sites through evolution. Several closely related species of yeast have been sequenced and comparison of the corresponding promoter regions has led to the discovery of conserved regulatory motifs [35,36]. Although lack of conservation does not imply non-functionality, significant conservation strongly argues for functionality of a putative regulatory element. To investigate this possibility, we performed a phylogenetic comparison to infer whether these sites are preserved among six sequenced *Saccharomyces *species using the PhyloGibbs program [37]. The program identified the **a**1-*α*2 binding site among a promoter set including many known haploid-specific genes (*HO, NEJ1, GPA1, STE4, *and *STE18*). This analysis also showed that the **a**1-*α*2 binding sites in the *RDH54, PDE1*and *MET31 *promoters are strongly conserved among multiple species, suggesting that these sites play an important functional role.

Our analysis identified a number of haploid-specific genes that do not appear to be direct targets of the **a**1-*α*2 repressor complex (Table 2). Genes in this list do not contain a recognizable **a**1-*α*2-binding site and, with the exception of *NEM1*, are not detectably bound by the **a**1-*α*2 complex in the ChIP assays. It is possible that **a**1-*α*2 indirectly turns off these genes by repressing an activator protein that is required for their expression. However, besides *MET31*, there were no obvious genes coding for activator proteins that were direct targets of the **a**1-*α*2 complex. It is possible that the haploid-specific genes without **a**1-*α*2 sites are indirectly repressed through more complex mechanisms that involve repression of *RME1*.

We also identified potential **a**1-*α*2-binding sites in the genome that do not appear to repress expression of nearby genes. Although sites from the *PRM8*, *PRM9*, *CDC25*, and *LSM1 *promoters appear to be moderate binding sites for the **a**1-*α*2 complex in vitro, ChIP and heterologous reporter assays showed these sites are neither bound by the proteins nor are functional repressor sites in vivo. Many of these sites lie in open reading frames of actively transcribed genes and so it is possible that transcription through the binding site or the chromatin structure of the region prevents high affinity binding by the complex. The model that the genomic context of these sites is important for their regulatory activity is further supported by our results that show that some of these sites, such as *COX13 *and *REX2*, function as strong **a**1-*α*2 dependent repressor sites in the context of the heterologous promoter. Although **a**1-*α*2 complex is bound to the *COX13 *site in vivo it does not appear to repress transcription of this gene in diploid cell. Interestingly, this binding site is very close to the end of the coding region of *IME4*, an inducer of meiosis that is expressed in diploid cells [38]. The *IME4 *gene is only expressed in diploid cells and it was thought that the **a**1-*α*2 complex may be indirectly activating its expression by repressing a repressor protein, such as *RME1*. However, the fact that **a**1-*α*2 binds to the downstream region of this gene suggests that it may play a direct role in its expression.

Our data shows that the algorithm we have developed is useful in sorting between direct and indirect targets of a transcription factor. Although we have used mutational data to define the binding site for the **a**1-*α*2 complex, in principal binding site sequences derived from site selection experiments may also be used. This analysis may also complement genome-wide ChIP studies to identify the target sites of the transcription factor.

## Conclusions

In summary, we show that combining microarray data with motif analysis, lets us distinguish between the genes that are direct targets of a transcription factor and those that are modulated because of secondary effects. We get excellent agreement of the computational predictions with location analysis by ChIP experiments. We find most of the direct targets of **a**1/*α*2 complex to be involved in the mating pathway, mating type switching, recombination and meiosis. We also found a few weak targets that are possibly involved in sensing and control of the metabolic state. We also see that the sites we predict solely based on single species data are often evolutionarily conserved in other species of *Saccharomyces.*

## Methods

### Combined scoring of genes from microarray data and mutational analysis

We define a scoring algorithm that ranks gene expression patterns. For gene *g*, the score is given in terms of the expression in different types of cells (**a**, *α *and **a**/*α*)

Score(*g*) = sgn((*X*_**a**_(*g*) + *X*_*α*_(*g*))/2 - *X*_**a**/*α*_(*g*)) [(*X*_**a**_(*g*)) + *X*_*α*_(*g*))/2 - *X*_**a**/*α*_(*g*)]^2 ^- *A*(*X*_**a**_(*g*) - *X*_*α*_(*g*))^2^.

We initially used the logarithm of expression level of the gene *g *for the three cell types for the variables *X*_t_(*g*) with t indicating the type. We have since found that using the complete expression data for the polyploids is a better strategy. In the final results, shown in the paper, *X*_**a**_(*g*) is the average of the log expression for **a**, **aa **and **aaa**. Likewise, *X*_*α *_(*g*) is the average from *α, αα *and *ααα*. *X*_**a**/*α*_(*g*) comes from averaging log expression over **aa***α*, **a***αα *and **aa***αα*. The polyploid averaged quantities tend to be less noisy (demonstrated, for example, by the quantities *X*_**a **_and *X*_*α *_being close to each other for the generic gene, which is not regulated by cell type. This, in turn, allows easier detection of genuine haploid-specific targets.

An explanation of the purpose served by different terms in the overall score is described below. The first term scores well when expression in diploids is lower than the average expression in haploids. The second term penalizes the gene if the expressions in different types of haploids are very different. *A *is chosen to be large enough so that known **a**-specific genes and *α*-specific genes score worse than known haploid-specific genes, but not large enough to overwhelm the first term. The optimal *A *is about 10. Comparison of the performance of our algorithm for *A *= 1 and *A *= 10, shows that the biologically known sites almost always stay near the top but further down in the list the second choice is better. The exception is a special gene: *MATα*1. Since *MATα*1 is not present in the *MAT****a ***type cell, there is a penalty for expression patterns. Cumulative probability for any gene to have higher score than a gene g is *P*_*exprn*_(*g*), namely, fraction of genes with score higher than g.

This scoring function would rank haploid-specific genes high but may not select out genes that are directly regulated by the **a**1-*α*2 repressor complex. In order to select for genes with an upstream region with a strong **a**1-*α*2 repressor, we used the binding site mutational data available [9]. In these experiments, repression of a heterologous promoter, incorporating single site mutations of a consensus binding site of the **a**1-*α*2 repressor, was measured. Under the assumption that the degree of repression is inversely proportional to how often that site is occupied, we derived the expression: 1/Repression ∝ [1 - 1/(1 + e^*βE*(*S*)^/*z*)] ≈ e^*βE*(*S*)^/*z *assuming near saturation of binding. The symbol *z *represents the fugacity and *β *is inverse of *k*_*B*_*T*, *k*_*B *_being the boltzman constant. The binding (free) energy is given by *E*(*S*) = Σ_*ib*_*ε*_*ib*_*S*_*ib*_, within the single base model [15,16]. The index *i *runs over the positions in the motif and *b *runs over the bases A, C, G, T.*S*_*ib *_is 1 or 0 depending upon whether the *i-*th base is *b *or not. The parameters *ε*_*ib *_represent effects of single base changes on the binding (free) energy. They are related to the weight matrix parameters [17,18] widely used to characterized variable motifs. Note that e^*βE*(*S*)^/*z *would more commonly be represented as (*K*/ [Protein])•exp(Δ*G*(*S*)/*RT*) in the biochemistry literature [18,39]. The independent base model is only an approximation and mutations in nearest neighbor sites could produce effects that we cannot estimate from the existing data. There is a better separation between well-known sites and generic sequences if an extra penalty is added to the score for neighboring base pairs which both differ from the consensus. In this way every base different from consensus and neighboring another base different from consensus draws an additional penalty to the binding energy score. This parameter was set to be ln(2), by experience. Although this method prevented many false positives, it also penalized a few genuine candidates, such as the binding sites in the promoters of *FAR1 *and *MATα*1. Thus, from the effect of the single base mutations, we estimated the parameters *ε*_*ib*_. Armed with these parameters, we found the probability *P*(*E*|*ε*, *L*) that a random sequence of a certain length, *L*, would have a subsequence of binding energy greater than *E*. For gene g, the strongest site in the upstream region of length *L *would have binding energy *E*_*g*_. Low values of *P*_*binding*_(*g*) = *P*(*E*_*g*_|*ε*,*L*), indicated the presence of a good binding site.

**Figure 5:**
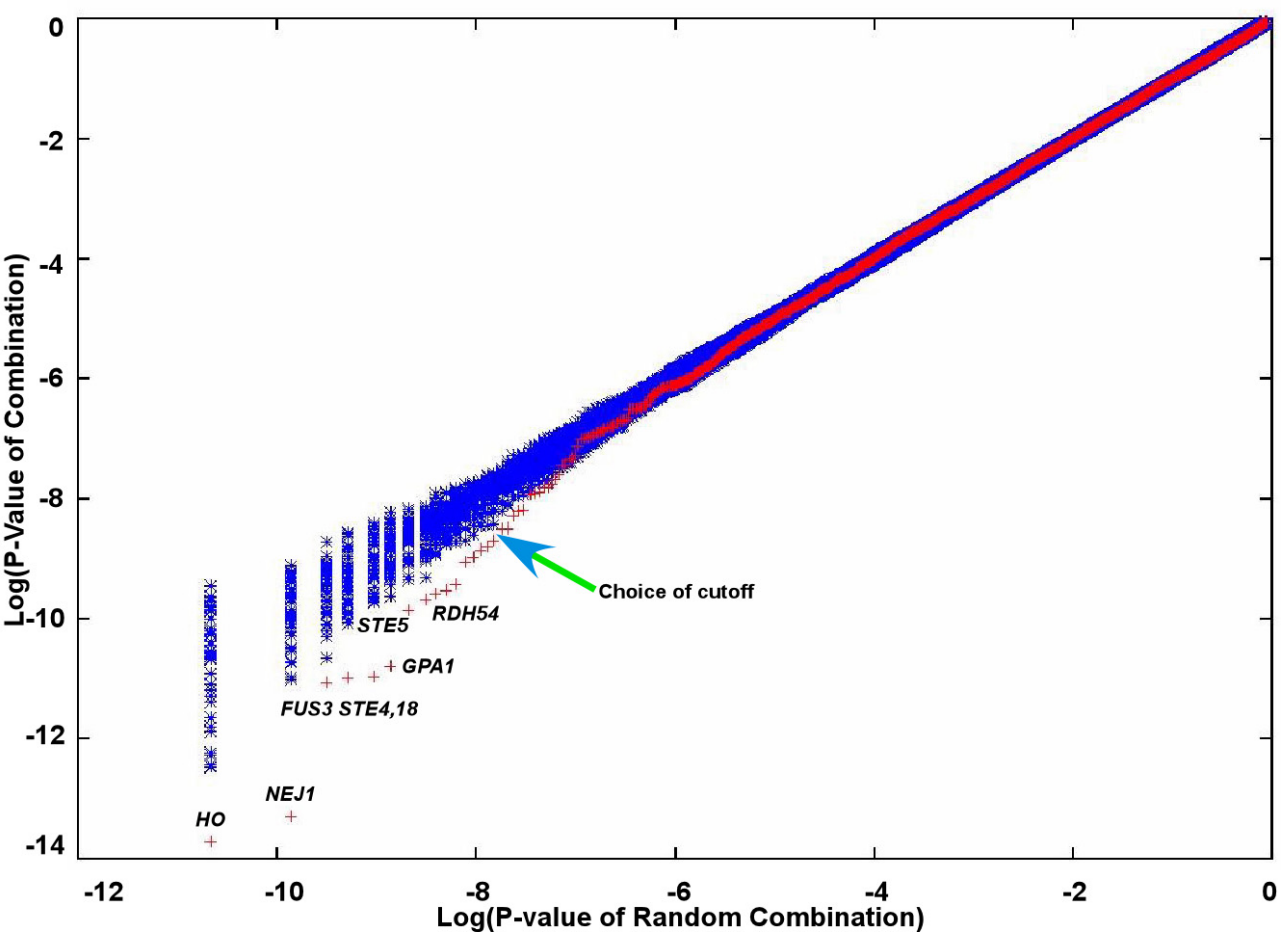
Significance of combined p-values. Natural logarithm of combined p-values for twenty permutations/scrambles generated, sorted and plotted (in blue) against average of log p-value. The genuine combined value is plotted in red.

The genes were ordered according to the lowest value of a combined p-value, *P*_*exprn*_(*g*) *P*_*binding*_(*g*), and then ranked as candidates for haploid-specific genes that are directly repressed by the **a**1-*α*2 complex. One of the issues in such studies is how to decide on how many of the top candidates are significant. This problem occurs even in solely, sequence based analysis as well [40]. In our study, we generated random combinations of expression p-values with scrambled binding p-values, so that we could choose a cutoff threshold by comparing the ordered p-values with the ordered "scrambled" p-values. Figure 5 plots these sorted p-values against average (log) sorted p-values for the random combinations. The comparison suggests that only about 10–15 top candidates in the list are significant.

### Weight matrix based search

A weight matrix [15-18] search for binding sites was performed using a set of known sites [9] to construct the matrix. Each matrix entry, *w*_*ia*_, was set to log((*f*_*ia *_+ *δ*)/(*P*_*a *_+ *δ*)), where *f*_*ia *_is the frequency with which base a appears in the *i*th position in the known sites, *P*_*a *_is the frequency with which base a appears in the promoters of genes and *δ *is a small number added to ensure that the weight matrix score is finite even when *f*_*ia *_= 0. Each subsequence, *S*_*ia*_, of length 20 in each promoter was assigned a weight matrix score Σ_*ia *_*S*_*ia *_*w*_*ia*_. After a threshold score is chosen, sites scoring above that threshold are declared to be binding sites.

### Automated primer generation

An automated procedure for generating primers flanking a specified site in the genome sequence, *σ*, was implemented. To each pair of numbers, *d*_*u*_, and *d*_*d*_, representing primer distances upstream and downstream of the candidate binding site respectively, and primer lengths *l*_*u*_, and *l*_*d*_, a score is assigned via

*S*(*d*_*u*_, *d*_*d*_, *l*_*u*_, *l*_*d*_,***σ***) = - Σ_*a*_*k*_*a*_(*P*_*a*_(*d*_*u*_, *d*_*d*_, *l*_*u*_, *l*_*d*_,***σ***) - )^2^

where the *P*_*a *_are functions including the melting temperatures, distances of upstream and downstream primers from the candidate site, total length of the bound region, and the difference between the primer melting temperatures. The  s are the preferred values of those functions. The *k*_*a *_are adjusted to reflect the relative importance of the parameters; for example it is more important that the difference in melting temperatures be close to zero than it is that the distance to the upstream primer match the distance to the downstream primer.

Values of *d*_*u*_, *d*_*d*_, *l*_*u *_and *l*_*d *_are restricted to those whose corresponding primers have GG, GC, CG, or CC at the end nearest the candidate site. Primers are identified by selecting the values of *d*_*u*_, *d*_*d*_, *l*_*u *_and *l*_*d *_which maximize *S*.

[A web-based interface to this algorithm is available at ]

### Chromatin immunoprecipitation

Chromatin immunoprecipitation (ChIP) was carried out as described previously [41] with the following modifications. One liter of JRY103 (*MATα/MAT****a****ade2-1/ADE2 HIS3/his3-11,15 leu2-3,112/leu2-3,112 trp1-1/trp1-1 ura3-1/ura3-1 ash1Δ::LEU2/ash1Δ::LEU2*) and JRY118 (*MATα/mat****a****Δ::TRP1 ade2-1/ADE2 HIS3/his3-11,15 leu2-3,112/leu2-3,112 trp1-1/trp1-1 ura3-1/ura3-1 ash1Δ::LEU2/ash1Δ::LEU2*) cultures were grown to an A600 of 0.5 and treated with 1% formaldehyde for 20 min at RT on a rotating shaker at low speed. Cells were collected, washed 2X with cold 1XTBS. Equal volumes of cells were aliquoted into ten 1.5 ml microfuge tubes, washed once with 1.5 ml of cold 1X TBS. The pellets in each tube were resuspended with 400 *μ*l of lysis buffer (50 mM HEPES, pH 7.4, 150 mM NaCl, 1 mM EDTA, 1% Triton X-100, 0.1% Na-Deoxycholate) plus 1 mM PMSF, 1 mM benzamidine, and 1X Protease inhibitor cocktail from Roche (Cat No. 1873580) and also manufacturer recommended concentration of protease inhibitor cocktail from SIGMA (Cat No., P 8215). To this 200 *μ*l of glass beads were added to each tube and lysed using a multitube vortexer at full speed for 30 min at 4°C. The lysate was transferred in a new tube and 400 *μ*l of lysis buffer was added and vortexed briefly. The lysates were centrifuged at 12,000 g for 10 min at 4°C and the supernatants were sonicated at 30% output for four 10 sec cycles with intermittent cooling on ice.

The lysates were cleared by centrifugation at 12,000 g for 10 min and 1 mM PMSF was added to the samples. A 1/10^th ^volume aliquot was removed and frozen to be used as total chromatin control. The remaining sample was precleared by the addition of 25 *μ*l recombinant protein G-agarose beads, incubated while nutating for 30 min and the supernatant was collected after centrifugation at 12,000 for 5 min. 1 *μ*l of rabbit anti-*α*2 antiserum (a gift from A. Johnson, UCSF) was added to each supernatant of the samples and incubated 12 h on a nutator at 4°C. To immunopreciptiate *α*2 50 *μ*l of recombinant protein G agarose beads (Roche) was added to the samples and nutated for 90 minutes at 4°C. The protein G beads were pelleted, washed once in low salt buffer (0.1%SDS, 1% Triton X-100, 20 mM Tris pH8.0, 2 mM EDTA and 150 mM NaCl), once in high salt (composition same as lowsalt + 500 mM NaCl), once in LiCl buffer (0.25 M LiCl, 1% IGEPAL, 1XTE and 1% Na-Deoxycholate) and twice with 1XTE (pH8.0). The immunoprecipitated DNA was eluted twice with 250 *μ*l of elution buffer (1%SDS and 0.1 M NaHCO3) and the eluates were pooled (500 *μ*l final volume). To this 20 *μ*l of 5 M NaCl was added and incubated 12 h at 65°C. To remove the crosslinks, 10 *μ*l of 0.5 M EDTA, 20 *μ*l of 1 M Tris-HCl, pH 7.5 and 2 *μ*l of proteinase K (10 mg/ml) was added and incubated for 45 minutes at 45°C. The DNA samples were extracted once with Phenol:chloroform:Isoamylalcohol and the DNA was ethanol precipitated, washed once with 70% ethanol and resuspended in 50 *μ*l (IP) or 500 *μ*l (TC) TE.

Purified DNA from the immunoprecipitated samples was subjected to multiplex PCR amplification with primers specific for the *STE6 *promoter as a positive control for the immunoprecipitation of *α*2 and the *YDL223C *ORF as a negative control for nonspecific immunoprecipitation, along with the specific primers for candidate *α*2-**a**1 target sites. PCRs were carried out in 50 *μ*l containing 10 pmols of each primer, 0.2 mM dNTPs, 2 mM MgCl2, 1X Eppendorf Taq buffer, 0.5X Taq Master buffer and 2.5 U of Eppendorf Taq polymerase. The amplifications were carried out at 94°C for 1 min and 30 secs, followed by 25 cycles of 94°C for 30 secs, 52°C for 1 min, and 72°C for 30 secs and a final extension step of 7 min at 72°C. The PCR products were separated on 2.5% agarose gels.

### Electrophoretic mobility shift assays

Oligonucleotides containing the predicted **a**1-*α*2 binding sites from within the ORFs of *URB1*, *PRM8*, *PRM9*, *YKL162C *and *CDC25 *and the promoters of *COX13, REX2, LSM1*, and *FMP14 *were synthesized, one strand was end-labeled with [*γ*-^32^P]-ATP, and then annealed with excess cold complementary oligonucleotide. The *HO(10*) and *HO(8*) **a**1-*α*2 sites within Upstream Regulatory Sequence 1 (URS1) of the *HO *promoter were used as strong and weak binding sites respectively. The EMSA was performed as described previously [42], using a constant 1.4 *μ*M **a**1 and five-fold titrations of *α*2 starting at 82 nM in protein dilution buffer (50 mM Tris pH 7.6. 1 mM EDTA, 500 mM NaCl, 10 mM 2-mercaptoethanol, 10 mg/ml bovine serum albumin).

### *β*-galactosidase assays

Oligonucleotides containing **a**1-*α*2 binding sites were synthesized with 5' overhangs to allow cloning into the *Xho*I site of pTBA23 (2*μ URA3 *Amp^r^), a reporter plasmid containing a *CYC1-lacZ *fusion [43]. Reporter constructs were transformed into JRY103 and JRY118 and the *β*-galactosidase activity was measured on three independent transformants, as described previously [14].

## List of Abbreviations

SAGE – Serial Analysis of Gene Expression

HD – Homeodomain

ORF – Open Reading Frame

ChIP – Chromatin immunoprecipitation

NHEJ – Non-Homologous End Joining

TC – Total Chromatin

IP – Immunoprecipitated sample

EMSA – Electrophoretic Mobility Shift Assay

PCR – Polymerase Chain Reaction

PMSF – Phenyl Methyl Sulfonyl Fluoride

EDTA – Ethylenediaminetetraacetic Acid

## Author's Contribution

VHN carried out the ChIP experiments, and wrote parts of the first draft of the manuscript. RAO did the bioinformatics analysis, and wrote programs facilitating primer design. ARB did EMSA and beta-gal assays. JRM constructed the strains and contributed to the design of the ChIP experiment. AKV and AMS supervised and coordinated the computational and the experimental research as well as prepared the manuscript. All authors contributed to the manuscript and approved the final version.
